# Resolving distinct molecular origins for copper effects on PAI-1

**DOI:** 10.1007/s00775-017-1489-5

**Published:** 2017-09-14

**Authors:** Joel C. Bucci, Carlee S. McClintock, Yuzhuo Chu, Gregory L. Ware, Kayla D. McConnell, Joseph P. Emerson, Cynthia B. Peterson

**Affiliations:** 1 Department of Biochemistry and Cellular and Molecular Biology, University of Tennessee, Walters Life Sciences Building, 1414 Cumberland Avenue, Knoxville, TN 37996 USA; 2 Department of Chemistry, Mississippi State University, Box 1115, Starkville, MS 39762 USA; 3 Department of Biological Sciences, A221 Life Sciences Annex, Louisiana State University, Baton Rouge, LA 70803 USA

**Keywords:** Protein structure, Calorimetry, PAI-1, Vitronectin, Somatomedin B domain, Copper

## Abstract

Components of the fibrinolytic system are subjected to stringent control to maintain proper hemostasis. Central to this regulation is the serpin plasminogen activator inhibitor-1 (PAI-1), which is responsible for specific and rapid inhibition of fibrinolytic proteases. Active PAI-1 is inherently unstable and readily converts to a latent, inactive form. The binding of vitronectin and other ligands influences stability of active PAI-1. Our laboratory recently observed reciprocal effects on the stability of active PAI-1 in the presence of transition metals, such as copper, depending on the whether vitronectin was also present (Thompson et al. Protein Sci 20:353–365, 2011). To better understand the molecular basis for these copper effects on PAI-1, we have developed a gel-based copper sensitivity assay that can be used to assess the copper concentrations that accelerate the conversion of active PAI-1 to a latent form. The copper sensitivity of wild-type PAI-1 was compared with variants lacking N-terminal histidine residues hypothesized to be involved in copper binding. In these PAI-1 variants, we observed significant differences in copper sensitivity, and these data were corroborated by latency conversion kinetics and thermodynamics of copper binding by isothermal titration calorimetry. These studies identified a copper-binding site involving histidines at positions 2 and 3 that confers a remarkable stabilization of PAI-1 beyond what is observed with vitronectin alone. A second site, independent from the two histidines, binds metal and increases the rate of the latency conversion.

**Electronic supplementary material:**

The online version of this article (doi:10.1007/s00775-017-1489-5) contains supplementary material, which is available to authorized users.

## Introduction

Plasminogen activator inhibitor-1 (PAI-1) is a serine protease inhibitor (serpin) responsible for controlling blood flow, and thus is subjected to strict regulation at the genetic as well as protein level [1]. PAI-1 inhibits tissue-type (tPA) and urokinase (uPA) plasminogen activators (PAs) [1]. Inhibition of the PAs limits the amount of activated plasmin at a site of injury or within the extracellular compartments, and in turn controls physiological processes including hemostasis [2], extracellular matrix turnover [3], and cell adhesive/migratory properties [4, 5]. Removal of PAI-1 results in compromised wound healing and mild bleeding states due to lack of control of the fibrinolytic components [6, 7], At the same time, excessive levels of PAI-1 result in atherosclerosis [8], fibrosis in several tissue types [9], inflammation [10], metabolic syndrome [11], and cancer [12].

**Fig. 1:**
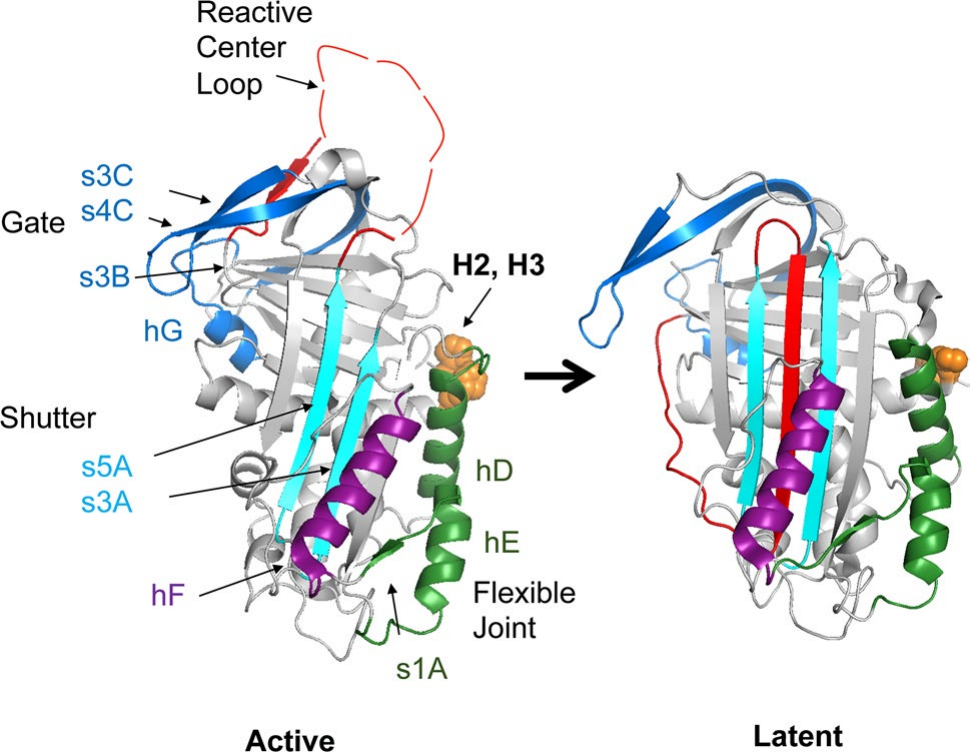
PAI-1 exists in an active and latent conformation. The structure of the active form of plasminogen activator inhibitor-1 (PAI-1) (PDB entry 3Q02) and the latent form of PAI-1 (PDB entry 1DVN) are shown. The reactive center loop (RCL) (*red*) acts as bait for proteases in the active form where it is solvent-exposed; it is inserted into central β-sheet A in the latent form. Several steps in the mechanism of the transition to the latent form have been proposed [31], and it appears that local unfolding within key regions is involved [30]. The shutter region (*teal*) expands, and the gate region loops (*blue*) rearrange during the RCL insertion that occurs during the latency conversion. Helix F (*purple*) may be displaced temporarily during unfolding of the hydrophobic core and/or rearrangement in the bottom half of the shutter when the RCL is added as an additional strand in the central β-sheet. The flexible joint region (*green*) is an important site for PAI-1 protein–protein interactions, including binding of the somatomedin B (SMB) domain [22]. The nomenclature for secondary structure is as follows: hA − hI are α-helices A − I, respectively; s1A is strand 1 of β-sheet A, s6B is strand 6 of β-sheet B, etc. The structure and location of histidines 2 and 3 are shown in *orange* for each view of the active or latent form of PAI-1

PAI-1 utilizes a solvent-exposed loop characteristic of serpins termed the reactive center loop (RCL) shown in Fig. 1 [13]. The RCL contains the same scissile bond that the PAs target in their natural substrate, plasminogen [13]. The inhibitory mechanism of PAI-1 mirrors the process of peptide bond cleavage by serine proteases until the final step, in which the acylated RCL inserts into the protein body, translocating the protease with a highly distorted active site to the opposite pole of the inhibitor [14]. Among serpins, PAI-1 is unique due to its inherent metastability in the active form [15]. PAI-1 has a half-life of approximately 1–2 h, then spontaneously undergoes a significant structural rearrangement to an inactive, latent form that can no longer inhibit proteases (Fig. 1) [16]. A directed sequence of steps accounts for this conversion to the latent structure, in which the uncleaved RCL must pass through the gate region loops (s3C-s4C, s3B-hG) before inserting in between the shutter region strands (s3A and s5A) [17–19]. For full RCL peptide insertion to occur, helix F (hF) covering the lower part of the shutter must be temporarily displaced [20]. An area denoted the flexible joint region (hD, hE, s1A) is important for protein–protein interactions, most notably with another glycoprotein, vitronectin (VN) [21]. VN binds to PAI-1 with high affinity (*K*_d_ ~0.1 nM) to localize and stabilize PAI-1 in the active form [22, 23].

Several factors influence the rate at which PAI-1 converts to the latent form, including ligands [24, 25], post-translational modifications [26], pH [27], and protein dynamics [28, 29]. Protein dynamics play a role in the latency process, whereby conditions that stabilize PAI-1 decrease protein dynamics, and those that destabilize PAI-1 increase protein dynamics [29]. Furthermore, hydrogen–deuterium exchange mass spectrometry (HDX-MS) has revealed local unfolding events within the hydrophobic core of PAI-1 that are hypothesized to be on the path to the latent state [30]. Ligands such as VN [24], antibodies [31], and RNA aptamers [25] stabilize PAI-1 to varying degrees; these ligands also restrict PAI-1 dynamics [28, 32].

Metal ion ligands such as copper have a particularly interesting effect on the stability of PAI-1, observed in its rate of latency conversion in the presence and absence of VN, as well as the isolated N-terminal somatomedin B (SMB) domain [28]. Copper binds to PAI-1 with high affinity (*K*_d_ ~0.09 μM), resulting in significant acceleration in the rate of latency conversion [33]. However, when bound to both VN (or the isolated SMB domain) and copper, PAI-1 is stabilized to an even greater extent than observed upon binding of VN or SMB alone [24]. These unusual metal effects have not been observed with any other serpins. HDX-MS experiments determined that addition of copper destabilizes PAI-1 by localized increases in protein dynamics [29], whereas the combination of copper and SMB binding results in decreases in dynamics within the same regions of PAI-1 [29].

Several parallels can be drawn comparing disease states due to dysregulation of PAI-1 and those due to copper homeostasis. Physiological copper concentrations are under stringent temporal and spatial control within and outside cells. Copper is a required cofactor for the function of many proteins, but in excess is highly toxic [34, 35]. Copper is integral to wound healing and angiogenesis, as its removal retards both processes [36]. Copper is known to be involved in vascular permeability, platelet–endothelial interactions, and the activation of smooth muscle cells [37]. On the other hand, elevated levels of copper can lead to oxidative damage including double strand DNA breaks, lipid peroxidation, and protein oxidation [34]. Although virtually all circulating copper is bound to proteins, namely ceruloplasmin or serum albumin [38], the concentrations of copper and PAI-1 within several biological compartments are such that physiologically relevant interactions can impact PAI-1 function [38–41].

This study investigates the molecular basis for copper effects on PAI-1 stability and overall function. Previous studies involving surface plasmon resonance with a nickel-NTA chip, stopped-flow binding measurements, and HDX-MS support a specific metal ion binding site within PAI-1 [24, 29, 33]. Several unanswered questions remain regarding copper effects on PAI-1: Where do copper ion(s) bind to PAI-1? What is the affinity and stoichiometry of PAI-1-copper interactions? How does copper increase PAI-1 stability (i.e., delay PAI-1 latency) when bound to PAI-1 in tandem with VN or the SMB domain? Alternatively, how does copper binding in the absence of vitronectin increase the rate of PAI-1 latency conversion? To investigate these questions, we have targeted histidine residues using site-directed mutagenesis and have compared copper binding and functional effects with wild-type PAI-1. We have utilized isothermal titration calorimetry (ITC) and gel-based assays to characterize metal-binding properties. Furthermore, we have used activity measurements and rates of latency conversion to determine whether the targeted histidines coordinate copper at a site that leads to the rapid loss of activity in PAI-1.

## Materials and methods

### Protein expression, purification and activity measurements

Wild-type PAI-1 and variants were cloned in a pET24d expression plasmid and expressed in Rosetta 2 DE3 pLysS *E. coli* cells. PCR-based site-directed mutagenesis was used to introduce mutations to the triplet codons encoding for histidines 2 and 3 and tryptophan 175 of PAI-1 to create H2A, H3A, H2AH3A, and H2AH3AW175F PAI-1 variants. Each of the mutations was confirmed by DNA sequencing. Wild-type PAI-1 and variant constructs were transformed into the expression cell line, grown, and expressed using established protocols [24]. A standard three-step purification scheme was employed [24], but with some notable adjustments for the variants, as follows. Cell lysis and the initial step of ion exchange chromatography were performed at pH 5.5 due to the change in pI when the N-terminal histidines were absent. This resulted in improved binding to the sulfopropyl column, and improved separation within the elutions. In addition, the N-terminal histidines are required for wild-type PAI-1 binding to an immobilized metal affinity chromatography column (IMAC). Since variants lacking the histidines failed to bind to an IMAC column, we used hydrophobic interaction chromatography with separation on a Phenyl Sepharose column instead. PAI-1 was dialyzed into 20 mM KH_2_PO_4_, 600 mM (NH_4_)_2_SO_4_, 1 mM EDTA, pH 7 overnight, loaded onto the Phenyl Sepharose column, and eluted with a linear reverse (NH_4_)_2_SO_4_ gradient (600–10 mM). In the final step of the purification, the protein was concentrated, and loaded on a size exclusion chromatography column as previously described [24].

The activity of wild-type and variant forms of PAI-1 was measured using standard assays [24]. PAI-1 was mixed with tPA at varying molar ratios ranging from 0.25 to 4 (PAI-1:tPA). The inhibitory capacity of PAI-1 was measured in a gel-based assay, wherein the PAI-1/tPA complex at each molar ratio was monitored and quantified by densitometry. PAI-1 inhibition of proteases was also measured in a kinetic assay in which the tPA-substrate (Spectrozyme tPA, Sekisui Diagnostics) was added to the mixture containing PAI-1 and tPA. Cleavage of the *p*-nitroanaline group of Spectrozyme tPA was detected by absorbance at 405 nm, allowing for the measurement of tPA activity over time at each molar ratio of PAI-1:tPA. Purified active wild-type PAI-1 fully inhibits tPA with 1.25 molar equivalents of PAI-1. Latent PAI-1 was generated by dilution in buffer (50 mM sodium phosphate, 300 mM NaCl, 1 mM EDTA, pH 6.25) to a 5 μM concentration, and incubation at 37 °C with light stirring for 6 days. The aforementioned activity measurements were performed to confirm that no active PAI-1 remained in the mixture.

### Metal ion titration gel assay

To determine the Cu(II) concentrations required to induce accelerated PAI-1 latency, we used a gel-based metal ion titration assay. Serial dilutions of CuSO_4_ were prepared, resulting in final Cu(II) concentrations ranging from 5 to 1000 μM. Addition of copper resulted in a drop of as much as 0.2 pH units, so the solution was readjusted using NaOH. Incubation with metal was initiated by adding PAI-1 at a final concentration of 4 μM and maintaining the mixture at 37 °C in 50 mM MOPS, 100 mM (NH_4_)_2_SO_4_ pH 7.4 for 30 min. After the 30-min incubation, PAI-1 was mixed in a 1:1 ratio with single-chain tPA (Molecular Innovations). Three control samples (PAI-1, tPA, and PAI-1/tPA) were also tested in which copper is not included. Non-reducing SDS-PAGE dye was added to each sample, followed by boiling for 10 min prior to loading on the gel. The free/latent PAI-1, free tPA, and PAI-1/tPA complex in each sample were separated and visualized by SDS-PAGE electrophoresis in a 4–12% gradient gel (Invitrogen). Samples were subjected to electrophoresis at 150 V for 75 min. SDS-PAGE gels were stained with Coomassie blue dye overnight, followed by three destaining steps of 90 min in deionized water containing 30% methanol and 7.5% glacial acetic acid. Gel pictures were captured using a ChemiDoc XRS molecular imager (Biorad), and gel band densitometry of the PAI-1/tPA complex bands was performed using Imagelab (Biorad). The band intensity of each sample was normalized to 100% with an untreated PAI-1/tPA complex sample, and to 0% using the gel background. Averages and error were generated (*n* = 2) for each assay, and the normalized band intensities were plotted as a function of the logarithm of total copper concentration. The midpoint copper concentration for wild-type PAI-1 or each variant was extrapolated from the semi-log plots as the copper concentration at which 50% of PAI-1/tPA complex was observed; this measurement was used as an expression of relative metal “sensitivity” of the various forms of PAI-1.

### Stability kinetics assays

To measure the half-life of PAI-1 latency conversion, stability assays were performed. PAI-1 at 0.1 μM was incubated in 50 mM MOPS, 100 mM (NH_4_)_2_SO_4_, 0.1 mM EDTA pH 7.4 at 37 °C. At specific time points, aliquots of PAI-1 were taken and mixed with 0.1 μM two-chain tPA (Molecular Innovations), in 50 mM MOPS, 100 mM (NH_4_)_2_SO_4_, 2 mM EDTA, 1% bovine serum albumin, pH 7.4. Individual samples were analyzed in 96-well plates by mixing with a final concentration of 1 mM Spectrozyme tPA substrate (Sekisui Diagnostics). The reaction was monitored over a 5-min time period via tPA substrate cleavage at the *p*-nitroanaline group that absorbs at 405 nm. This measurement was conducted in triplicate for multiple incubation times that covered a range up to ten times the PAI-1 half-life. The averages of the reaction slopes were entered into GraphPad Prism Software for analysis. The reaction slopes were plotted as a function of PAI-1 incubation time to calculate tPA activity over time. Taking the negative slope and normalizing the data (100% at *t* = 0; 0% at the lower baseline time points) yielded the normalized PAI-1 activity as a function of PAI-1 incubation time. To determine the rate of latency conversion (*k*_lat_), the data were fit to the exponential decay equation *x*_*t*_ = *x*_0_e^−*kt*^, where *x*_*t*_ is the amount of active PAI-1 at a given time, *x*_0_ is the amount of PAI-1 at time 0, *k* is the rate of latency conversion, and *t* is time. The half-life of latency conversion was determined as ln(2) *k* ^−1^. For assays involving copper, PAI-1 was incubated in the same buffer with 215 μM CuSO_4_ added. The buffer was corrected for pH change due to copper addition with NaOH. For assays involving the SMB domain, a 1:2 PAI-1:/SMB complex was formed prior to incubation at 37 °C or copper addition. Controls confirmed that there was no effect of copper directly on tPA activity [i.e., substrate cleavage in the presence of copper was the same as in the absence of metal].

### Isothermal titration calorimetry

Isothermal titration calorimetry (ITC) was used to determine the thermodynamic parameters associated with Cu(II) interactions with PAI-1. Active and latent forms of wild-type and variants of PAI-1 were dialyzed into 100 mM MOPS, 250 mM (NH_4_)_2_SO_4_ at 10 °C for 2 h. Because of the relative instability of the H2AH3A variant of PAI-1, our studies on H2AH3A PAI-1 utilized a variant that contained an additional mutation, W175F. The W175F switch is a well characterized substitution used to stabilize the active form of PAI-1 during purification and to ensure that a significant amount of latent H2AH3A PAI-1 did not accumulate during the ITC experiment [42]. Residue 175 is not proximal to the N terminus and is not involved in metal ion coordination or its subsequent effects on PAI-1 stability. Copper solutions were prepared by diluting CuSO_4_ into the matched dialysate buffer. The copper stock concentration was verified using atomic absorbance spectroscopy, and the pH corrected to 7.4 at 10 °C using NaOH. Twenty micromolar PAI-1 (2.2 mL total volume) and copper solution (4 mL) were degassed under vacuum for 10 min at 5 °C prior to loading into the (Malvern) MicroCal VP-ITC. The PAI-1 solution was loaded into the ITC cell (1.394 mL), and the copper solution was loaded into the ITC syringe. The reference and experimental ITC cells were equilibrated to 10 °C prior to the start of the experiment. After a preliminary injection of 2 µL, each additional injection consisted of a 4 µL injection at 120 or 240 s intervals. Experiments were performed at least in duplicate. Control experiments measuring dilution heats were performed in which the copper solution was injected into buffer. The data were baseline corrected using NITPIC software (University of Texas, Southwestern), and fit to either a single-site or two-site non-symmetric binding model in SEDPHAT (National Institutes of Health). The data and fits were represented using GUSSI software (University of Texas, Southwestern).

## Results

### PAI-1 variants exhibit altered metal sensitivities

The N terminus of PAI-1 comprises a consensus copper binding motif similar to sites that have been characterized in serum albumin [43, 44], superoxide dismutase [45], and prion protein (PrP^c^) [46, 47]. This motif contains two histidines, H2 and H3, which are thought to be involved in binding copper. To test for differences in copper binding, we generated three variants in which the N-terminal histidines were replaced with alanine residues, denoted as H2A, H3A, and H2AH3A PAI-1. Whereas wild-type PAI-1 can be purified using an immobilized metal affinity column [24], variants lacking these histidines did not bind to a nickel-charged metal affinity column, providing direct evidence that these residues are assessable and can coordinate metal ions.

**Fig. 2:**
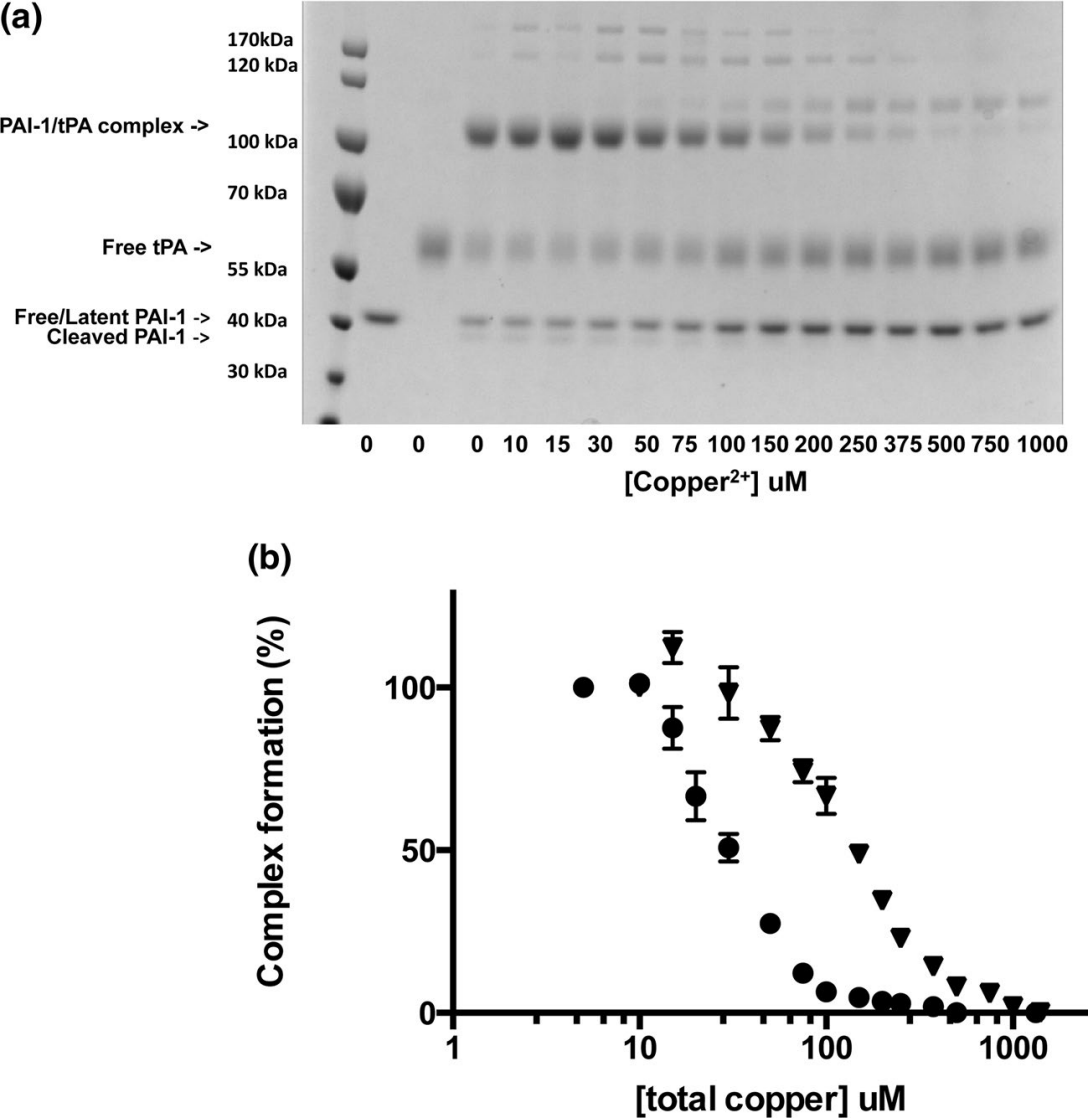
Copper titration gel assay for wild-type PAI-1. **a** Equimolar mixtures of PAI-1 and tissue-type plasminogen activator (tPA) were prepared after 30 min incubations of PAI-1 with Cu(II) at a wide range of concentrations (5–1000 μM, total). *Lane 1* contains a molecular ladder, *lanes 2*–*4* are controls in which PAI-1, tPA, and PAI-1/tPA, respectively, were incubated without Cu(II). Each subsequent lane contains PAI-1/tPA mixtures incubated with varying Cu(II) concentrations, as indicated. Densitometry of the PAI-1/tPA complex (series of bands above the 100 kDa band) within the gel was quantified using ImageLab. PAI-1 incubated without copper (100%) (*lane 3*) and gel background (0%) were used to normalize each data point as a percentage. **b** PAI-1/tPA complex formation is plotted as a function of the total Cu(II) concentration. The metric for comparison of wild-type PAI-1 to PAI-1 variants is taken as the midpoint in copper concentration (“sensitivity”) for which 50% PAI-1/tPA complex is observed in the densitometry data. Equimolar mixtures of PAI-1 and tPA were prepared after 30 min incubations of PAI-1 with varying Cu(II) concentrations (10–1000 μM, total) in 50 mM MOPS, 100 mM (NH_4_)_2_SO_4_ pH 7.4 at 37 °C. Gel densitometry of the PAI-1/tPA complex was quantified using ImageLab. The band intensity of each sample was normalized to 100% with an untreated PAI-1/tPA complex sample, and to 0% using the gel background. The percent of PAI-1/tPA complex formation is plotted as a function of the total Cu(II) concentration. Wild-type and H2AH3A PAI-1 are represented by *inverted triangles* and *circles*, respectively. Experiments were performed in duplicate

While this result clearly establishes that a metal-binding site exists near the N terminus of PAI-1, the key question is to determine whether copper binding effects the transition between active and latent forms of PAI-1. Initially, a gel-based copper sensitivity titration assay was developed to determine the copper concentrations that invoke an accelerated rate of latency conversion on PAI-1. For this assay, PAI-1 samples were incubated with Cu(II) concentrations of 5–1000 μM for 30 min, and then mixed with tPA. The samples were separated by non-reducing SDS-PAGE. After staining, the band intensities of the PAI-1/tPA complex at each copper concentration were quantified using gel densitometry. At low concentrations of Cu(II), PAI-1 exhibits robust complex formation with tPA, indicating that it remains in an active form (Fig. 2a). At intermediate copper concentrations, there is clearly a mixture of inactive PAI-1 and the PAI-1/tPA complex, and at high metal concentrations, all PAI-1 is latent and no PAI-1/tPA complex is observed. The data yield a sigmoidal curve in a semi-log plot, in which the midpoint of the transition represents the effective concentration of copper that accelerates the latency transition (Fig. 2b). The midpoint in the curve for wild-type PAI-1 is approximately 150 μM Cu(II) and serves as a benchmark “sensitivity” for comparison to variants lacking the hypothesized copper binding residues (Table 1). This result indicates considerably weaker “sensitivity” in promoting PAI-1 latency compared to the affinity previously estimated for copper binding to PAI-1 using stopped-flow methods (*K*_d_ ~90 nM) [33], which presented a discrepancy that was investigated further.

**Table 1:** Copper sensitivities of wild-type and variant forms of PAI-1 determined from gel titration assays

PAI-1 Variant	Copper sensitivity, µM
Wild-type	150
H2A	20
H3A	20
H2AH3A	20
Wild-type + SMB	200
H2AH3A + SMB	200
Wild-type + 250 mM (NH_4_)_2_SO_4_	>1000
H2AH3A + 250 nM (NH_4_)_2_SO_4_	>1000

A variant lacking the putative copper-binding residues that promote latency would exhibit decreased sensitivity compared with wild-type PAI-1, and would require higher Cu(II) concentrations to accelerate PAI-1 latency. Thus, if copper coordination by the histidine residues accounts for the accelerated rate of PAI-1 latency, then we predict that the copper sensitivity of these variants would be decreased. In contrast, we observed the opposite effect, in which variants lacking the N-terminal histidines are more sensitive to copper compared with wild-type PAI-1 (Fig. 2b). Each of the single variants (H2A, H3A) as well as the double variant (H2AH3A) of PAI-1 required lower Cu(II) concentrations to induce latency, with midpoints in the sensitivity curves of approximately 20 μM Cu(II) (Table 1). This unusual observation indicates that the accelerated latency of PAI-1 in the presence of copper is not due to binding to the N-terminal histidines. This result also points to the possibility of multiple metal-binding sites with different affinities and different effects on the latency transition.

A caveat should be noted because these gel titrations are not equilibrium measurements and the “sensitivities” are not simple binding constants. Instead, this assay captures points across a time course with samples that contain a mixture of free PAI-1 and the PAI-1-copper complex. At the incubation time of 30 min, a mixture of active and latent PAI-1 is present, with copper-bound and copper-free portions of each form, as determined by the *K*_d_ for copper binding and *t*_1/2_ values for latency of PAI-1 and the PAI-1-copper complex. Because PAI-1-copper complexes may have different half-lives for the latency transition [24], wild-type and variant forms of PAI-1 can be reliably compared with this method only when the *t*_1/2_ values of the copper-bound and copper-free species are similar for the wild-type and engineered forms of PAI-1. Fortunately, because the H2A and H3A amino acid substitutions did not influence latency conversion kinetics, we could directly compare the copper sensitivities among these variants of PAI-1.

### SMB domain binding dampens copper sensitivity of PAI-1

**Fig. 3:**
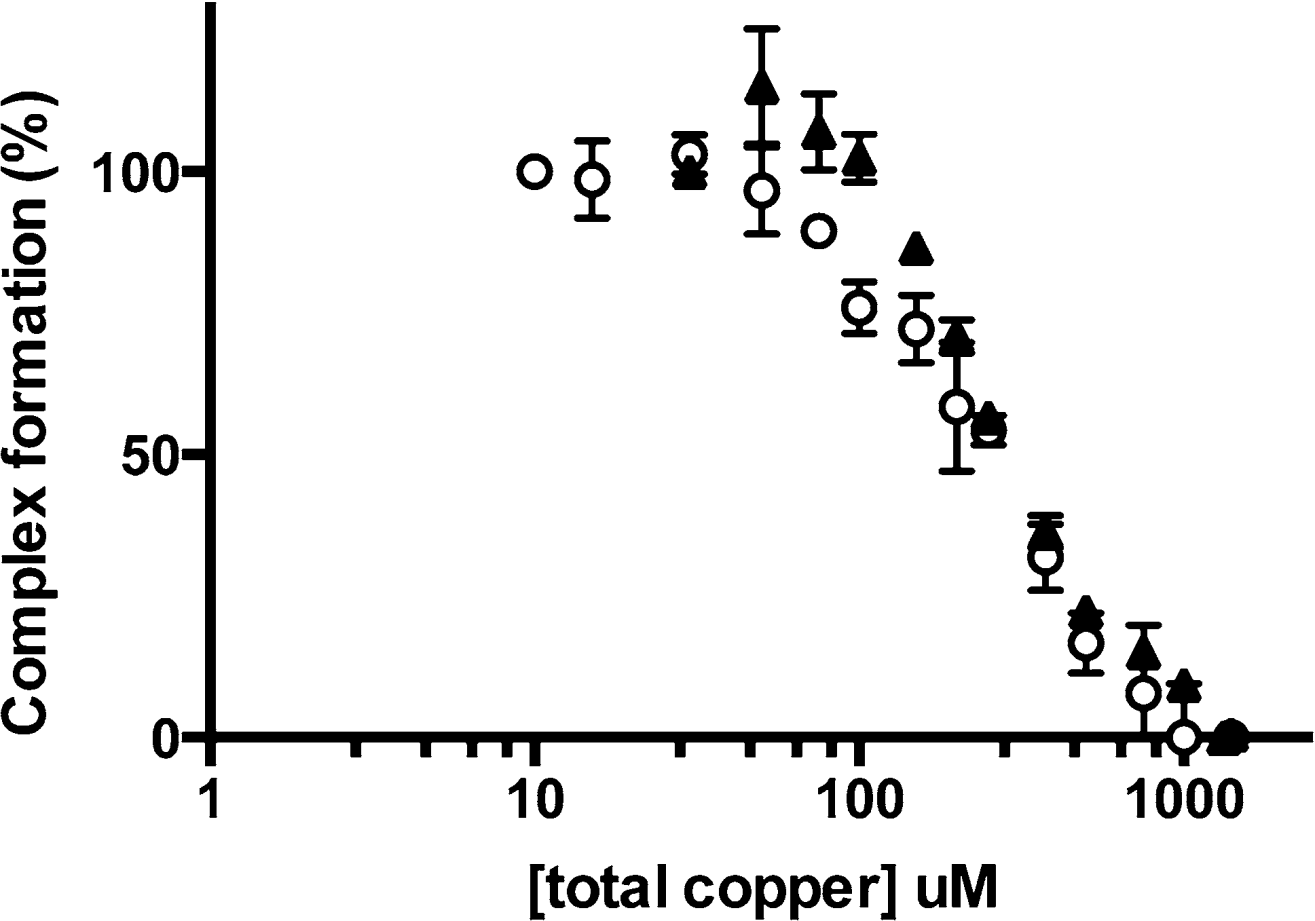
Copper titration comparing wild-type PAI-1 versus H2AH3A PAI-1. Equimolar mixtures of PAI-1 and tPA were prepared after 30 min incubations of PAI-1 with varying Cu(II) concentrations (10–1000 μM, total) in 50 mM MOPS, 100 mM (NH_4_)_2_SO_4_ pH 7.4 at 37 °C. Gel densitometry of the PAI-1/tPA complex was quantified using ImageLab. PAI-1 incubated without copper (100%) and gel background (0%) were used to normalize each data point as a percentage. The percent of PAI-1/tPA complex formation is plotted as a function of the total Cu(II) concentration. Wild-type and H2AH3A PAI-1 are represented by *triangles* and *circles*, respectively. Experiments were performed in duplicate

The SMB domain of VN binds within the flexible joints region of PAI-1, resulting in a modest stabilization of the active form [21, 24]. Using the gel-based copper titration assay, we tested for the effect of SMB binding to PAI-1 on the copper sensitivity range for accelerated latency. We expected that the formation of the PAI-1/SMB complex would lead to decreased copper sensitivity, based on the stabilizing effects of SMB binding in the absence of copper ions. Wild-type PAI-1 combined with the SMB domain was indeed less sensitive to copper, demonstrated by a midpoint in the gel titration assay of approximately 200 μM Cu(II) (Fig. 3; Table 1). Prior work has established that the SMB domain does not interact with copper [29], so the sensitivity measured in this assay is solely due to copper binding to PAI-1. For comparison, we also tested H2AH3A PAI-1 combined with the SMB domain, and observed a similar copper sensitivity. While these results represent a modest decrease in copper sensitivity upon binding of SMB to wild-type PAI-1, they reveal a much more significant decrease for H2AH3A PAI-1. The SMB domain exhibits high affinity binding to PAI-1 (*K*_d_ ~1 nM) [21], and the micromolar concentrations of proteins used in these assays are well in excess of the *K*_d_. Although it is possible that there are somewhat different binding affinities for wild-type and H2AH3A PAI-1, the similar dramatic effects observed in this gel-based assay indicate that both are fully saturated with SMB under these conditions. Since binding of the SMB domain alters latency kinetics independent of copper effects, comparisons with these data alone are to be taken with caution in this non-equilibrium assay. Nonetheless, it is clear that binding of the SMB domain to both wild-type PAI-1 and the H2AH3A variant dampens sensitivity to copper so that differences in the rate of the latency transition that arise due to the histidine replacements are not observed.

### Copper stabilizes the PAI-1/SMB complex through binding to the N-terminal site

**Fig. 4:**
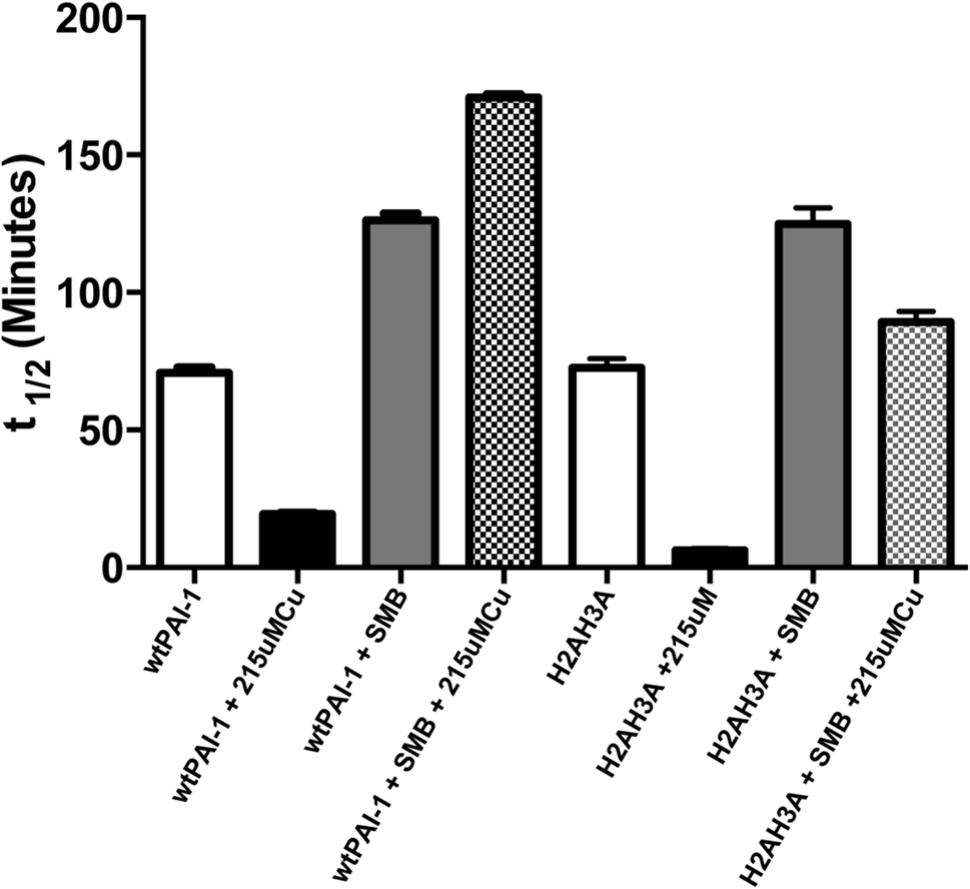
Effects of copper and/or SMB binding on stability kinetics measurements of wild-type and H2AH3A PAI-1. Wild-type or H2AH3A PAI-1 were incubated at 0.1 μM in 50 mM MOPS, 100 mM (NH_4_)_2_SO_4_, 0.1 mM EDTA, pH 7.4 at 37 °C. Four conditions were tested: PAI-1 alone (*white bars*), PAI-1 + 215 μM Cu(II) (*black bars*), PAI-1 + SMB domain (*gray bars*), and PAI-1 + SMB domain + Cu(II) (*checkered bars*). At various time points, PAI-1 is mixed with 0.11 μM tPA to react with all PAI-1 remaining in the active form. tPA activity is measured by addition of 1 mM (final) Spectrozyme tPA substrate and the absorbance of substrate cleavage is monitored at A405 for 5 min. PAI-1 inhibitory activity is plotted versus incubation time and fit to an exponential decay function to determine the rate of latency transition. Experiments were performed in triplicate

To determine how the binding of copper to N-terminal histidines affects the rate of PAI-1 latency, we measured the latency conversion kinetics of these variants in the presence and absence of copper and the SMB domain (Fig. 4; Table 2). We first reproduced our previous studies on wild-type PAI-1, but instead used a MOPS buffer system, which offers the benefit of decreased Cu(II)–buffer interaction compared with Tris buffer used previously [24]. Wild-type PAI-1 remains in the active form for a half-life of 70 min in the MOPS buffer. Ligand binding in MOPS buffer imparts the same effects on PAI-1 as in Tris buffer; thus wild-type PAI-1 in the presence of Cu(II) (215 μM) undergoes latency significantly faster, with a half-life of 20 min. As observed previously, wild-type PAI-1 is modestly stabilized by incubation with the SMB domain of VN, with a half-life for the latency conversion of 125 min. In the presence of copper and the SMB domain, PAI-1 is further stabilized, with a half-life of 170 min.

**Table 2:** Kinetic properties for copper binding to wild-type and H2AH3A PAI-1

PAI-1 variant	*t*_1/2_ for latency conversion, min	*t*_1/2_ for latency conversion [+Cu(II)], min	*t*_1/2_ for latency conversion (+SMB), min	*t*_1/2_ for latency conversion [+Cu(II), +SMB], min
Active WT	70.7 (±3.6)	19.4 (±0.8)	126.2 (±4.3)	170.9 (±2.1)
Active H2AH3A	72.7 (±5.6)	6.2 (±1.0)	125.0 (±1 10.0)	89.2 (±6.7)

Next, we tested the H2AH3A PAI-1 variant under the same conditions to assess the role of copper binding to these residues in the PAI-1 latency conversion (Fig. 4; Table 2). We measured the rate of latency of the H2AH3A variant alone to determine how the N-terminal histidine substitutions themselves influence the latency transition independent of copper effects. In these assays, H2AH3A PAI-1 has a half-life of latency conversion of 74 min, which is not statistically different from wild-type PAI-1, indicating that the introduction of these amino acid substitutions has no intrinsic effect on the rate of the latency transition in PAI-1. In addition, H2AH3A PAI-1 is destabilized in the presence of copper, with a half-life for the latency conversion of 6 min, a shorter half-life than observed with wild-type PAI-1 bound to copper. This more pronounced rate of acceleration induced by copper binding to the variant compared to wild-type PAI-1 agrees well with the increased copper sensitivity of the H2AH3A variant observed in the gel titration assay. This result clearly indicates that a second metal-binding site remains in the H2AH3A variant of PAI-1 and suggests that this additional site is responsible for the accelerated latency conversion. Furthermore, since the half-life for the latency transition is actually shorter with the H2AH3A PAI-1 variant, the increased latency differential for this variant relative to wild-type PAI-1 points to a potential stabilizing role of copper binding to the N-terminal histidines.

Based on results from the gel titration assay (Fig. 2b), we hypothesized that copper binding to the N-terminal histidines is the source of the enhanced stabilization observed when copper is incubated with the PAI-1/SMB complex. Whereas the copper titration gel assay with the H2AH3A PAI-1 variant indicated that metal binding to these two histidines was not the interaction that promotes that latency conversion, the assay with the H2AH3A PAI-1/SMB complex in fact revealed a pronounced decrease in sensitivity equivalent to that observed with the wild-type PAI-1/SMB complex. Consistently, latency conversion kinetics indicated that H2AH3A PAI-1 bound to SMB domain is stabilized to the same extent as wild-type PAI-1, with a half-life of latency conversion of 125 min (Fig. 4; Table 2). Interestingly, however, the H2AH3A PAI-1/SMB complex was not further stabilized compared to wild-type PAI-1 by the binding of copper, with a half-life of latency conversion of only 89 min. This result stands in contrast to observations of the wild-type PAI-1/SMB complex behavior and supports our hypothesis, indicating that the enhanced stabilization of the PAI-1/SMB complex is imparted through copper binding to the N-terminal histidines of PAI-1. Thus, one of the most unusual and striking features we observed in our metal studies with PAI-1, i.e., that copper binding concurrently with the SMB domain yields a highly stable form of PAI-1 that converts to latency more slowly than with the SMB domain alone [24, 29, 33], can be attributed to metal coordination by these N-terminal histidines.

### ITC identifies differences in Cu(II) interactions with active, latent and variant forms of PAI-1

Isothermal titration calorimetry (ITC) proved to be an important tool for investigating the thermodynamic characteristics of Cu(II) binding to PAI-1. This method offers the advantage of providing a full thermodynamic characterization for the interaction, with affinity and stoichiometry explicitly determined, along with binding enthalpies. In the ITC experiments, copper was titrated from a stock solution via small injections into a thermal cell containing PAI-1, and the power needed to maintain the sample temperature was measured. To maintain the active form of PAI-1 over the time course of the experiment, we lowered the experimental temperature to 10 °C, and increased the (NH_4_)_2_SO_4_ concentration to 250 mM. The increased (NH_4_)_2_SO_4_ protects against non-specific metal ion effects, and was shown in gel titration assays (Figure 1S) to render PAI-1 relatively insensitive to metal effects on the latency transition. It should be noted that the PAI-1 latency transition itself results in no observable heat changes in control ITC experiments [48].

**Fig. 5:**
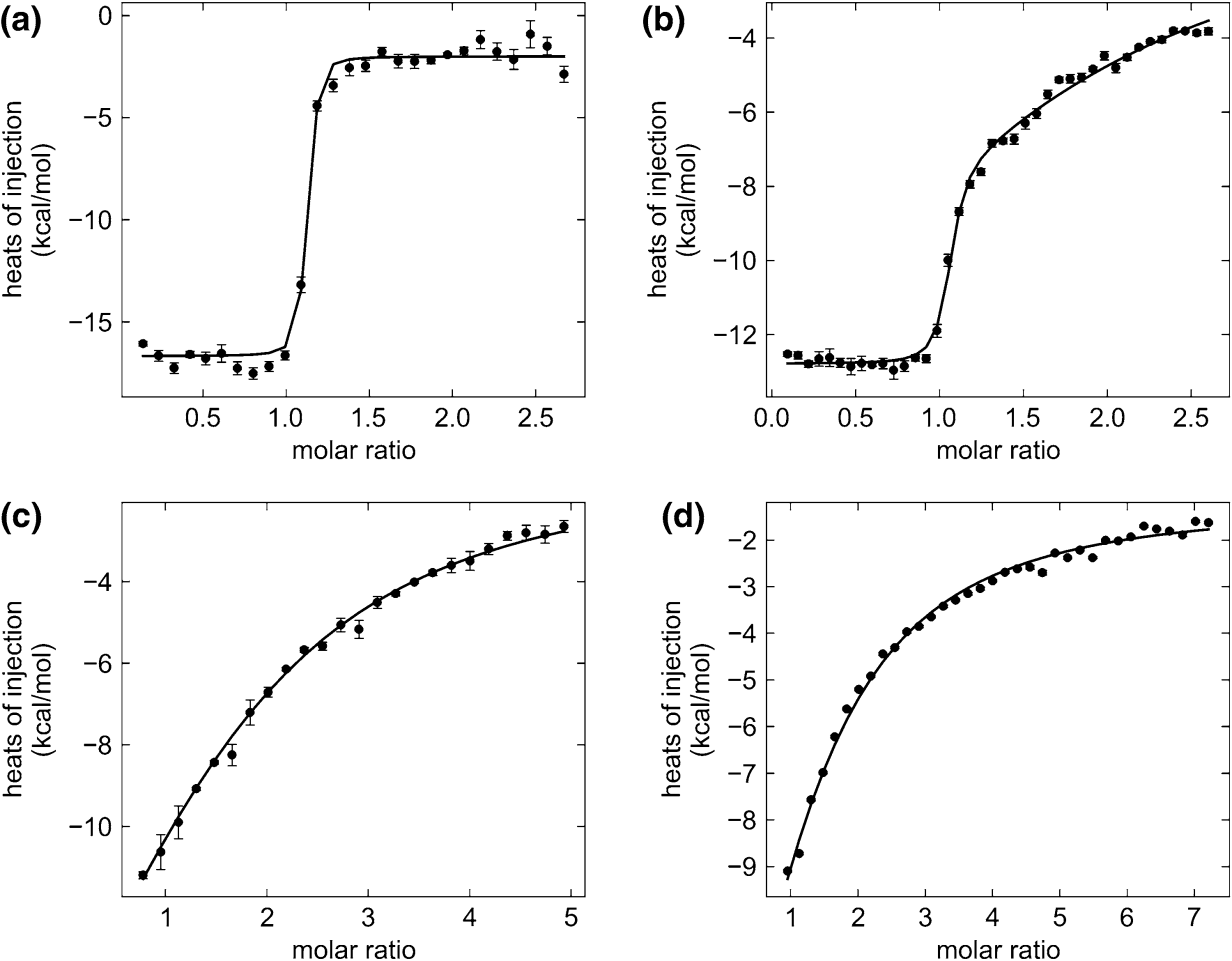
Isothermal titration calorimetry of active, latent and variant forms of PAI-1. Representative isotherms are shown for the titration of **a** active wild-type PAI-1, **b** latent wild-type PAI-1, **c** active H2AH3A PAI-1, **d** latent H2AH3A PAI-1 with Cu(II). Isotherms for the full data set for copper titration into active wild-type PAI-1, including global fitting with replicates of four concentrations of Cu(II) titration solutions, are shown in Figure 2S. Isotherms for the full data set for copper titration into latent wild-type PAI-1, including global fitting for replicates of three concentrations of Cu(II) titration solutions, are shown in Figure 3S. Isotherms for the full data set for copper titration into active H2AH3A PAI-1, including global fitting for replicates of two concentrations of Cu(II) titration solutions, are shown in Figure 4S. Isotherms for the full data set for copper titration into latent H2AH3A PAI-1, including global fitting for replicates of two concentrations of Cu(II) titration solutions, are shown in Figure 5S. The data are baseline corrected in NITPIC software, and fit to a one-site binding model in SEDPHAT software. The data and fit are represented in GUSSI software via heats of injection (kcal mol^−1^) as a function of copper/PAI-1 molar ratio. Experiments were performed in triplicate

Titrations of active wild-type PAI-1 with Cu(II) ranging up to 650–800 μM were performed and the data were subject to global fitting analysis. We observed one sharp transition with an *n* value of 1.2 in all cases, so the data were fit to a single-site binding model. Fitting of replicate data yielded an average *K*_obs_ value of 5.4 × 10^7^ M^−1^ and Δ*H*_obs_ = −16.2 kcal mol^−1^ (Fig. 5a, Figure 2S; Table 3). This corresponds to an effective *K*_d_ = 19 nM, where the *K*_d_ = 1/*K*_obs_, and averages for Δ*G*_obs_ and −*T*Δ*S*_obs_ equal to −10.0 and 5.9 kcal mol^−1^, respectively. These results agree well with prior analyses of PAI-1-Cu(II) binding using stopped-flow kinetics that determined a *K*_d_ of ~ 90 nM [33], and give evidence that copper binding to the active wild-type PAI-1 complex is driven by a highly favorable change in enthalpy with a slightly unfavorable change in entropy penalty. By contrast, latent wild-type PAI-1 binds Cu(II) with two distinct binding transitions, one tight binding transition accompanied by a significantly weaker one. Global fits to multiple data sets indicated that latent PAI-1 binding of Cu(II) fits best to a two-site non-symmetric binding model, yielding average *K*_obs_ terms of 2.4 × 10^7^ and 3.4 × 10^4^ M^−1^, and Δ*H*_obs_ terms of −12.0 and −11.2 kcal mol^−1^ for the high and low affinity binding sites, respectively (Fig. 5b, Figure 3S; Table 3). These parameters correspond to average *K*_d_ values of 42 nM and 30 μM for the high and low affinity sites, respectively. The high affinity Cu(II) binding event in latent wild-type PAI-1 shows similar free energy to that in the active PAI-1 described above, although the enthalpic and entropic contributions are different compared with the site in the active form. Interestingly, the weaker binding site has a similar Δ*H*_obs_ term to the high affinity site, yet its −*T*Δ*S*_obs_ term is more destabilizing, which effectively weakens the Cu(II) binding affinity to this site (Table 3). The observed thermodynamic parameters are a good benchmark for comparison within this study, but further analysis and deconvolution of the data from these complex ion equilibria are required to identify the origins of these changes.

**Table 3:** Thermodynamic properties for Cu(II) binding to wild-type and H2AH3A PAI-1 at 10 °C

PAI-1 variant	Model (*n*)	[Cu(II)]	*K*_obs_, M^−1^	*K*_d_^a^	Δ*G*_obs_^b^, (kcal/mol)	Δ*H*_obs,_ (kcal/mol)	−*T*Δ*S*_obs_^c^, (kcal/mol)
Active WT	1	650 µM	7.6 (±3.1) × 10^8^	13 (±9) nM	−10.2 (±0.3)	−15.9 (±0.3)	5.7 (±0.3)
Active WT	1	700 µM	8.2 (±4.9) × 10^7^	19 (±10) nM	−10.0 (±0.7)	−16.4 (±0.6)	6.3 (±0.7)
Active WT	1	750 µM	4.8 (±5.5) × 10^7^	20 (±20) nM	−9.9 (±0.4)	−15.9 (±1.2)	5.0 (±1.2)
Active WT	1	800 µM	4.1 (±2.8) × 10^7^	24 (±12) nM	−9.8 (±0.2)	−16.4 (±0.6)	6.5 (±0.6)
Latent WT	2	650 µM High affinity	2.2 (±0.4) × 10^7^	46 (±10) nM	−9.5 (±0.1)	−12.0 (±2.8)	2.5 (±0.1)
		low affinity	4.20 (±2.8) × 10^4^	24 (±12) μM	−6.0 (±0.6)	−11.2 (±3.0)	5.1 (±0.6)
Latent WT	2	750 µM High affinity	2.6 (±0.7) × 10^7^	38 (±10) nM	−9.6 (±0.2)	−12.0 (±2.9)	2.4 (±0.2)
		Low affinity	2.9 (±1.1) × 10^4^	35 (±12) μM	−5.8 (±0.4)	−11.2 (±3.1)	5.4 (±0.2)
Active H2AH3A	1	1200 µM	4.2 (±0.9) × 10^4^	24 (±5) μM	−6.0 (±0.1)	−20.0 (±12.0)^d^	14 (±12)^d^
Active H2AH3A	1	1350 µM	2.7 (±2.1) × 10^4^	37 (±12) μM	−5.7 (±0.4)	−51.6 (±13.0)^d^	46 (±13)^d^
Latent H2AH3A	1	1050 µM	5.2 (±4.0) × 10^4^	19 (±15) μM	−6.1 (±0.6)	−37.5 (±11.0)^d^	31 (±11)^d^
Latent H2AH3A	1	1200 µM	7.7 (±4.6) × 10^4^	13 (±6) μM	−6.3 (±0.4)	−16.4 (±0.6)^d^	10 (±0.4)^d^

^a^ *K*_d_ calculated from the following relationship: *K*_d_ = 1/*K*_obs_

^b^Δ*G*_obs_ calculated from Δ*G*_obs_ = −RT ln *K*_obs_

^c^−*T*Δ*S*_obs_ term calculated from Δ*G*_obs_ = Δ*H*_obs_ − *T*Δ*S*_obs_

^d^Parameters measured at the low sensitivity limit of the ITC and likely have significant error associated with them

For comparison, the PAI-1 variant housing the H2AH3A substitutions was also studied via ITC. As anticipated, Cu(II) titrations on active and latent forms of H2AH3A PAI-1 revealed significant differences compared to wild-type PAI-1 binding of copper. Only a single binding event was observed for both latent and active forms of this variant. Superficially, this binding event resembles the weak second binding event observed in the latent wild-type PAI-1. The thermodynamic parameters extracted from the Cu(II) binding isotherm of the active H2AH3A PAI-1 were an average *K*_obs_ = 3.4 × 10^4^ M^−1^ and Δ*H*_obs_ value of −35.8 kcal mol^−1^ (Fig. 5c, Figure 4S; Table 3). This corresponds to an average *K*_d_ value of 30.5 μM. This value agrees well with the gel assay result in which the PAI-1 “sensitivity” midpoint value for the latency conversion was 20 μM (Fig. 2b), which is similar to the affinity the weak binding site in the latent wild-type protein. Although we have good faith in the accuracy of the *K*_obs_ and Δ*G*_obs_ terms from this series of ITC experiments, the Δ*H*_obs_ term reported here is likely inaccurate [49]. The Δ*H*_obs_ term is primarily determined from the first injections of the experiment, and when the affinity of this binding isotherm is low there is limited curvature in the isotherm, which leads to an inaccuracy in the measured enthalpy term. Additional experiments are required to assess the impact of the Δ*H*_obs_ for this system. The absence of the tight binding event within H2AH3A PAI-1 suggests that the higher-affinity site can be attributed to the N-terminal histidines of PAI-1. The latent H2AH3A PAI-1 exhibits similar copper binding compared to the active H2AH3A variant. Latent H2AH3A PAI-1 binds Cu(II) with a single binding event, with an average *K*_obs_ = 6.4 × 10^4^ M^−1^and Δ*H*_obs_ value of −27.0 kcal mol^−1^ (Fig. 5d, Figure 5S; Table 3). Similar to the active variant described above, the Δ*H*_obs_ term for the latent H2AH3A PAI-1 is likely inaccurate. At this level of analysis, we consider the copper binding events to active and latent H2AH3A PAI-1 to be indistinguishable, where they both yield weak binding events with highly similar binding constants.

## Discussion

### Connecting copper binding to PAI-1 and its functional stability as an active inhibitor

Hydrogen deuterium exchange measurements showed that copper binding to PAI-1 increases protein dynamics in regions relevant to the latency process [29]. PAI-1 lacking the N-terminal histidines retains the copper effects on protein dynamics, which are localized to the same regions as in wild-type PAI-1 [29]. This is further evidence that copper binding to the N-terminal domain cannot be solely responsible for the accelerated latency transition, and that the other metal-binding site in PAI-1 may be involved in this process.

The goal of this study was to explore the effects that copper invokes on the stability of active PAI-1 [24, 33] as observed previously with the metal dependence in rates of the latency conversion and changes in dynamics [29]. The data collected for the H2AH3A variant of PAI-1 identified the histidine-rich, N-terminal region of PAI-1 to be involved in copper binding. A comparison of the properties of wild-type PAI-1 and the H2AH3A variant (summarized in Tables 1, 2, 3) establish that H2AH3A PAI-1 is more sensitive to latency conversion, and indeed converts faster in the presence of saturating levels of Cu(II) compared with wild-type PAI-1. Our ITC results demonstrate that there is a single, high-affinity Cu(II) binding site in active PAI-1 (*K*_d_ 19 nM), whereas there are two copper-binding sites in latent PAI-1 (*K*_d_ values were measured to be 40 nM and 40 μM). Copper binding to the high affinity site in active PAI-1 was also characterized using stopped-flow kinetics, yielding a *K*_d_ ~90 nM [33] and demonstrating agreement between the two techniques. On the other hand, the gel-based copper titrations reveal “sensitivities” in latency conversion that can be attributed to the second, weaker copper binding site. Substitutions for the N-terminal histidines perturbed the high affinity copper binding site in active PAI-1, and this variant protein had an accelerated rate of latency.

Combining these results, we conclude that the high affinity, N-terminal copper binding domain of PAI-1 plays a role in chelating free Cu(II) in solution, effectively protecting the active form of the protein, and the low affinity copper binding site promotes conversion to the latent form of PAI-1. Upon conversion from the active to the latent form, major structural changes occur in PAI-1, where two distinctive metal-binding sites are present. Structural differences in the latent form involve the insertion of the RCL into the central β-sheet, which displaces helices D and E in the flexible joint region and moves them away from the N terminus (Fig. 1). Based on the heat of Cu(II) coordination, it is likely that the latent form of PAI-1 contains different copper^−^coordinating ligands to complement the N-terminal histidines and/or to comprise the second weaker site. In H2AH3A PAI-1, there is no high affinity site to bind adventitious Cu(II), which then leads this variant to sampling conformations more similar to the latent form which are ultimately highly sensitive to copper. These differences affect the results collected for wild-type and variant forms of PAI-1 in the gel assays. Another possibility is that the high salt conditions used for the ITC experiments may disrupt copper binding to the weaker metal-binding site in the active protein. This notion is supported by the gel assays at matching salt concentrations, in which the stability of wild-type or H2AH3A PAI-1 was desensitized to Cu(II) over a broad concentration range (10 to >1000 μM). While specific disruption of the weaker metal-binding site is conceivable, the effect of higher ammonium sulfate could also be a more general effect, consistent with the known effects of ammonium sulfate solutions on protein stabilization.

### Copper coordination by the N-terminal histidines stabilizes the PAI-1/SMB complex

We have reported previously that wild-type PAI-1 in complex with the SMB domain of VN is further stabilized by copper binding [24]; however, H2AH3A PAI-1 does not exhibit the synergistic effects of copper and SMB binding. Thus, it appears that Cu(II) binding to the N-terminal histidines is responsible for the stabilizing effect of metal in combination with PAI-1 binding by the SMB domain. The stabilizing effects on PAI-1 that result from binding of the SMB domain alone are attributed to binding in the flexible joints region, resulting in widespread restriction of dynamics, especially in nearby helices D, E, and F [28]. Copper binding to the PAI-1/SMB complex results in a restriction of dynamics near the N terminus of PAI-1, a phenomenon that does not occur in the absence of SMB domain [29]. This restriction of dynamics likely influences a local unfolding event on the proximal hA that occurs on a time scale relevant to the latency conversion process [30].

## Conclusions

Copper binds to the N-terminal histidines of PAI-1 (H2, H3), but binding to this site does not result in accelerated latency in the presence of copper. In fact, binding of copper to this site stabilizes the active form of PAI-1. We propose that a second, low-affinity copper binding site is responsible for the copper-related destabilization of PAI-1. While this second site remains to be identified, it appears that the different structures of active and latent PAI-1 allow for alternative Cu(II) coordination sites. Potential Cu(II) binding residues near the N-terminal histidines in active PAI-1, including E81 and H364, will be a focal point of future studies. Whether the weaker, destabilizing Cu(II)-coordination site represents an alternative binding mode in active PAI-1 or a distinct, independent site also must be a focal point of future studies. While the N-terminal histidines do not constitute the binding site that promotes the latency transition, this site is valuable in mediating the striking synergistic effects between copper binding and the SMB domain. Interestingly, histidine-rich, N-terminal copper- and nickel-binding domains have been recently identified in numerous proteins, but a specific role for these metal-binding motifs is unclear [50–53]. It is clear, however, that this N-terminal site in PAI-1 is critical because it appears to stabilize the active conformation of PAI-1 with SMB present when metal ions would otherwise quickly render PAI-1 in its latent form, which is ineffective as an inhibitor.

## Electronic supplementary material

Below is the link to the electronic supplementary material.

Supplementary material 1 (PDF 1630 kb)

## Abbreviations

HDX: Hydrogen–deuterium exchange
HDX-MS: Hydrogen–deuterium exchange coupled to mass spectrometry
IMAC: Immobilized metal affinity chromatography
IPTG: Isopropyl β-d-1-thiogalactopyranoside
ITC: Isothermal titration calorimetry
H2A, H3A, and H2AH3A-PAI-1: Mutant form of PAI-1 lacking N-terminal histidines at positions 2, 3, or 2 and 3 in the amino acid sequence, respectively
RCL: Reactive center loop
SMB: Somatomedin B domain
PAs: Plasminogen activators
tPA: Tissue-type plasminogen activator
uPA: Urokinase plasminogen activator
VN: Vitronectin
PAI-1: Plasminogen activator inhibitor-1
Serpin: Serine protease inhibitor

## References

[1] Dellas C, Loskutoff DJ (2005). Historical analysis of PAI-1 from its discovery to its potential role in cell motility and disease. Thromb Haemost.

[2] Podor TJ, Campbell S, Chindemi P, Foulon DM, Farrell DH, Walton PD, Weitz JI, Peterson CB (2002). Incorporation of vitronectin into fibrin clots. Evidence for a binding interaction between vitronectin and gamma A/gamma′ fibrinogen. J Biol Chem.

[3] Silverman GA, Whisstock JC, Bottomley SP, Huntington JA, Kaiserman D, Luke CJ, Pak SC, Reichhart JM, Bird PI (2010). Serpins flex their muscle: I. Putting the clamps on proteolysis in diverse biological systems. J Biol Chem.

[4] Minor KH, Peterson CB (2002). Plasminogen activator inhibitor type 1 promotes the self-association of vitronectin into complexes exhibiting altered incorporation into the extracellular matrix. J Biol Chem.

[5] Garg N, Goyal N, Strawn TL, Wu J, Mann KM, Lawrence DA, Fay WP (2010). Plasminogen activator inhibitor-1 and vitronectin expression level and stoichiometry regulate vascular smooth muscle cell migration through physiological collagen matrices. J Thromb Haemost.

[6] Carmeliet P, Schoonjans L, Kieckens L, Ream B, Degen J, Bronson R, De Vos R, van den Oord JJ, Collen D, Mulligan RC (1994). Physiological consequences of loss of plasminogen activator gene function in mice. Nature.

[7] Fay WP, Garg N, Sunkar M (2007). Vascular functions of the plasminogen activation system. Arterioscler Thromb Vasc Biol.

[8] Vaughan DE (2005). PAI-1 and atherothrombosis. J Thromb Haemost.

[9] Ghosh AK, Vaughan DE (2012). PAI-1 in tissue fibrosis. J Cell Physiol.

[10] Juhan-Vague I, Alessi MC, Mavri A, Morange PE (2003). Plasminogen activator inhibitor-1, inflammation, obesity, insulin resistance and vascular risk. J Thromb Haemost.

[11] Alessi MC, Juhan-Vague I (2006). PAI-1 and the metabolic syndrome: links, causes, and consequences. Arterioscler Thromb Vasc Biol.

[12] Andreasen PA, Egelund R, Petersen HH (2000). The plasminogen activation system in tumor growth, invasion, and metastasis. Cell Mol Life Sci.

[13] Lawrence DA, Olson ST, Palaniappan S, Ginsburg D (1994). Serpin reactive center loop mobility is required for inhibitor function but not for enzyme recognition. J Biol Chem.

[14] Olson ST, Swanson R, Day D, Verhamme I, Kvassman J, Shore JD (2001). Resolution of Michaelis complex, acylation, and conformational change steps in the reactions of the serpin, plasminogen activator inhibitor-1, with tissue plasminogen activator and trypsin. Biochemistry.

[15] Dupont DM, Madsen JB, Kristensen T, Bodker JS, Blouse GE, Wind T, Andreasen PA (2009). Biochemical properties of plasminogen activator inhibitor-1. Front Biosci (Landmark Ed).

[16] Stout TJ, Graham H, Buckley DI, Matthews DJ (2000). Structures of active and latent PAI-1: a possible stabilizing role for chloride ions. Biochemistry.

[17] Kvassman JO, Verhamme I, Shore JD (1998). Inhibitory mechanism of serpins: loop insertion forces acylation of plasminogen activator by plasminogen activator inhibitor-1. Biochemistry.

[18] Dupont DM, Blouse GE, Hansen M, Mathiasen L, Kjelgaard S, Jensen JK, Christensen A, Gils A, Declerck PJ, Andreasen PA, Wind T (2006). Evidence for a pre-latent form of the serpin plasminogen activator inhibitor-1 with a detached beta-strand 1C. J Biol Chem.

[19] Hagglof P, Bergstrom F, Wilczynska M, Johansson LB, Ny T (2004). The reactive-center loop of active PAI-1 is folded close to the protein core and can be partially inserted. J Mol Biol.

[20] Gettins PG (2002). The F-helix of serpins plays an essential, active role in the proteinase inhibition mechanism. FEBS Lett.

[21] Zhou A, Huntington JA, Pannu NS, Carrell RW, Read RJ (2003). How vitronectin binds PAI-1 to modulate fibrinolysis and cell migration. Nat Struct Biol.

[22] Jensen JK, Wind T, Andreasen PA (2002). The vitronectin binding area of plasminogen activator inhibitor-1, mapped by mutagenesis and protection against an inactivating organochemical ligand. FEBS Lett.

[23] Stoop AA, Lupu F, Pannekoek H (2000). Colocalization of thrombin, PAI-1, and vitronectin in the atherosclerotic vessel wall: A potential regulatory mechanism of thrombin activity by PAI-1/vitronectin complexes. Arterioscler Thromb Vasc Biol.

[24] Thompson LC, Goswami S, Ginsberg DS, Day DE, Verhamme IM, Peterson CB (2011). Metals affect the structure and activity of human plasminogen activator inhibitor-1. I. Modulation of stability and protease inhibition. Protein Sci.

[25] Madsen JB, Dupont DM, Andersen TB, Nielsen AF, Sang L, Brix DM, Jensen JK, Broos T, Hendrickx ML, Christensen A, Kjems J, Andreasen PA (2010). RNA aptamers as conformational probes and regulatory agents for plasminogen activator inhibitor-1. Biochemistry.

[26] Bager R, Johansen JS, Jensen JK, Stensballe A, Jendroszek A, Buxbom L, Sorensen HP, Andreasen PA (2013). Protein conformational change delayed by steric hindrance from an N-linked glycan. J Mol Biol.

[27] Mangs H, Sui GC, Wiman B (2000). PAI-1 stability: the role of histidine residues. FEBS Lett.

[28] Trelle MB, Hirschberg D, Jansson A, Ploug M, Roepstorff P, Andreasen PA, Jorgensen TJ (2012). Hydrogen/deuterium exchange mass spectrometry reveals specific changes in the local flexibility of plasminogen activator inhibitor 1 upon binding to the somatomedin B domain of vitronectin. Biochemistry.

[29] Bucci JC, Trelle MB, McClintock CS, Qureshi T, Jorgensen TJ, Peterson CB (2016). Copper(II) ions increase plasminogen activator inhibitor type 1 dynamics in key structural regions that govern stability. Biochemistry.

[30] Trelle MB, Madsen JB, Andreasen PA, Jorgensen TJ (2014). Local transient unfolding of native state PAI-1 associated with serpin metastability. Angew Chem Int Ed Engl.

[31] Egelund R, Schousboe SL, Sottrup-Jensen L, Rodenburg KW, Andreasen PA (1997). Type-1 plasminogen-activator inhibitor—conformational differences between latent, active, reactive-centre-cleaved and plasminogen-activator-complexed forms, as probed by proteolytic susceptibility. Eur J Biochem.

[32] Trelle MB, Dupont DM, Madsen JB, Andreasen PA, Jorgensen TJ (2014). Dissecting the effect of RNA aptamer binding on the dynamics of plasminogen activator inhibitor 1 using hydrogen/deuterium exchange mass spectrometry. ACS Chem Biol.

[33] Thompson LC, Goswami S, Peterson CB (2011). Metals affect the structure and activity of human plasminogen activator inhibitor-1. II. Binding affinity and conformational changes. Protein Sci.

[34] Jomova K, Valko M (2011). Advances in metal-induced oxidative stress and human disease. Toxicology.

[35] Uriu-Adams JY, Keen CL (2005). Copper, oxidative stress, and human health. Mol Asp Med.

[36] Finney L, Vogt S, Fukai T, Glesne D (2009). Copper and angiogenesis: unravelling a relationship key to cancer progression. Clin Exp Pharmacol Physiol.

[37] Schuschke DA (1997). Dietary copper in the physiology of the microcirculation. J Nutr.

[38] Linder MC, Hazegh-Azam M (1996). Copper biochemistry and molecular biology. Am J Clin Nutr.

[39] Kramer ML, Kratzin HD, Schmidt B, Romer A, Windl O, Liemann S, Hornemann S, Kretzschmar H (2001). Prion protein binds copper within the physiological concentration range. J Biol Chem.

[40] Osterberg R (1980). Physiology and pharmacology of copper. Pharmacol Ther.

[41] Hartter DE, Barnea A (1988). Brain tissue accumulates 67copper by two ligand-dependent saturable processes. A high affinity, low capacity and a low affinity, high capacity process. J Biol Chem.

[42] Jensen JK, Thompson LC, Bucci JC, Nissen P, Gettins PG, Peterson CB, Andreasen PA, Morth JP (2011). Crystal structure of plasminogen activator inhibitor-1 in an active conformation with normal thermodynamic stability. J Biol Chem.

[43] Zhang Y, Wilcox DE (2002). Thermodynamic and spectroscopic study of Cu(II) and Ni(II) binding to bovine serum albumin. J Biol Inorg Chem.

[44] Jing M, Liu R, Yan W, Tan X, Chen Y (2016). Investigations on the effects of Cu(2+) on the structure and function of human serum albumin. Luminescence.

[45] Danielsson J, Awad W, Saraboji K, Kurnik M, Lang L, Leinartaite L, Marklund SL, Logan DT, Oliveberg M (2013). Global structural motions from the strain of a single hydrogen bond. Proc Natl Acad Sci USA.

[46] Burns CS, Aronoff-Spencer E, Dunham CM, Lario P, Avdievich NI, Antholine WE, Olmstead MM, Vrielink A, Gerfen GJ, Peisach J, Scott WG, Millhauser GL (2002). Molecular features of the copper binding sites in the octarepeat domain of the prion protein. Biochemistry.

[47] Gogineni DP, Spuches AM, Burns CS (2015). Calorimetric investigation of copper binding in the N-terminal region of the prion protein at low copper loading: evidence for an entropically favorable first binding event. Inorg Chem.

[48] Boudier C, Gils A, Declerck PJ, Bieth JG (2005). The conversion of active to latent plasminogen activator inhibitor-1 is an energetically silent event. Biophys J.

[49] Turnbull WB, Daranas AH (2003). On the value of c: can low affinity systems be studied by isothermal titration calorimetry?. J Am Chem Soc.

[50] Melino S, Santone C, Di Nardo P, Sarkar B (2014). Histatins: salivary peptides with copper(II)- and zinc(II)-binding motifs. Perspecgtives for biomedical applications, FEBS J.

[51] Harford C, Sarkar B (1997). Amino terminal Cu(II)- and Ni(II)-binding (ATCUN) motif of proteins and peptides: metal binding, DNA cleavage, and other properties. Acc Chem Res.

[52] Haas KL, Puterman AB, White DR, Thiele DJ, Franz KJ (2011). Model peptides provide new insights into the role of histidine residues as potential ligands in human cellular copper acquisition via Ctr1. J Am Chem Soc.

[53] Miyamoto T, Fukino Y, Kamino S, Ueda M, Enomoto S (2016). Enhanced stability of Cu^2+^-ATCUN complexes under physiologically relevant conditions by insertion of structurally bulky and hydrophobic amino acid residues into the ATCUN motif. Dalton Trans.

