# Supplementary material for: Resolving distinct molecular origins for copper effects on PAI-1
Source: J Biol Inorg Chem. 2017 Sep 14;22(7):1123–35. doi: 10.1007/s00775-017-1489-5 (PMC5613068; doi:10.1007/s00775-017-1489-5)

Resolving Distinct Molecular Origins for Copper Effects on PAI-1

Supplemental Information

Joel C. Bucci,[1,3] Carlee S. McClintock,[1] Yuzhuo Chu, [3] Gregory L. Ware, [3] Kayla D. McConnell,[2] Joseph P. Emerson, [2] and Cynthia B. Peterson,[1,3,∫]

[1]Department of Biochemistry and Cellular and Molecular Biology, Walters Life Sciences Building, 1414 Cumberland Avenue, University of Tennessee, Knoxville, TN 37996, USA

[2]Department of Chemistry, Box 1115, Mississippi State University, Mississippi State, MS 39762, USA

[3]Department of Biological Sciences, A221 Life Sciences Annex, Louisiana State University, Baton Rouge, LA 70803, USA

[,∫]To whom correspondence should be addressed: cbpeterson@lsu.edu, FAX: 225-578-8825

<u>Figure 1S</u>: Copper Sensitivity Measurements in Gel Assays on Wild-type and H2AH3A PAI-1 in the Presence of High Salt,

Equimolar mixtures of PAI-1 and tPA were prepared after 30 minute incubations of PAI-1 with copper(II) at a wide range of copper(II) concentrations (10-1000 µM, total) 50 mM MOPS, pH 7.4 at 37 °C, containing 250 mM $(NH_4)_2SO_4$ instead of the standard concentration of 100 mM $(NH_4)_2SO_4$ used for these assays (*e.g.* in <u>Figure</u> 2). Gel densitometry and data analysis were performed as described under Methods. The percent of PAI-1/tPA complex formation is plotted as a function of the total Cu(II) concentration. Wild-type and H2AH3A PAI-1 are represented by squares and open triangles, respectively. Experiments were performed in duplicate.

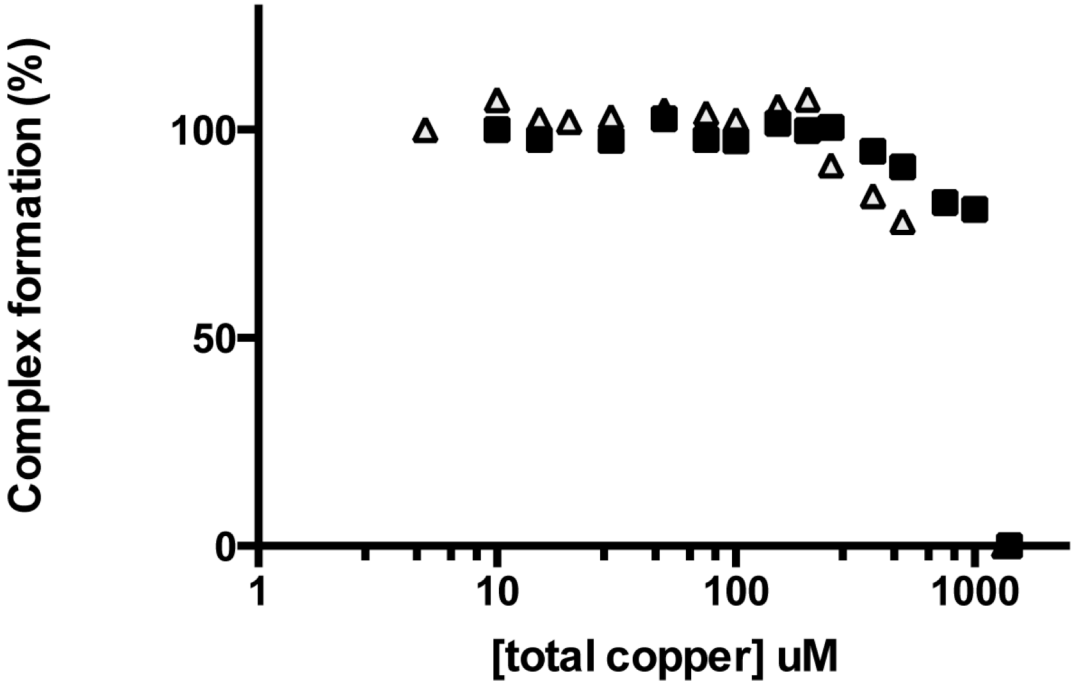

Figure 2S: ***ITC Measurements using Active Wild-Type PAI-1***
Active wild-type PAI-1 was buffer exchanged into 100 mM MOPS, 250 mM $(NH_4)_2SO_4$, pH 7.4 at 10 °C using a PD10 column (GE Healthcare), followed by dialysis for 2 hours. A stock copper(II) solution concentration was verified using atomic absorption spectroscopy. Copper(II) solutions were made from the ITC buffer dialysate, and then pH corrected to 7.4. Protein and copper(II) solutions were degassed with spinning for 10 minutes prior to loading (PAI-1 at 20 μM, copper(II) 600-900 μM) into the 1.394 mL ITC cell, and syringe, respectively. Copper(II) from the syringe entered the cell in 4 μL injections, at 240 second intervals, totalling 30 injections. PAI-1 with 650 μM copper(II) datasets are shown with black circles, squares, and inverted triangles. PAI-1 with 700 μM datasets are shown with light gray triangles, and diamonds. PAI-1 with 750 μM copper(II) datasets are shown with gray stars, and crosses. PAI-1 with 800uM copper(II) datasets are shown with light gray squares, inverted triangles, and circles. The data were baseline corrected in NITPIC software, and a global fit to all of the data was performed using a one-site binding model in SEDPHAT software. The data, global fit and residuals are represented in GUSSI software via heats of injection (*kcal/mol*) as a function of copper(II)/PAI-1 molar ratio.

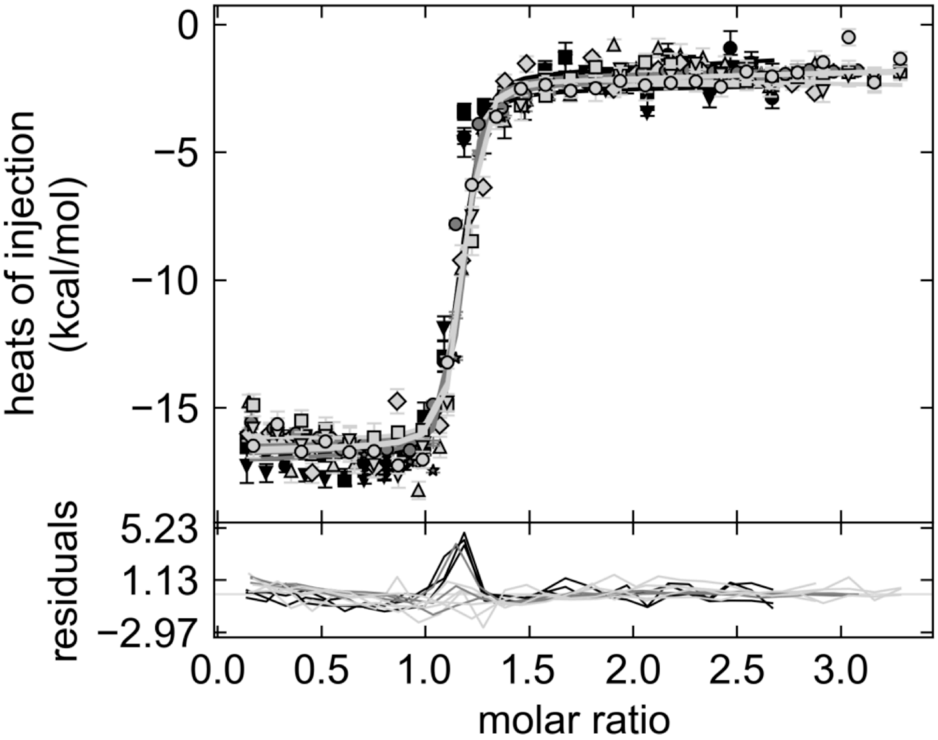

<u>Figure 3S</u>: ***ITC Measurements using Latent Wild-Type PAI-1***
Latent wild-type PAI-1 was buffer exchanged into 100 mM MOPS, 250 mM (NH4)2SO4, pH 7.4 at 10 °C using a PD10 column (GE Healthcare), followed by dialysis for 2 hours. A stock copper(II)solution concentration was verified using atomic absorption spectroscopy. Copper(II)solutions were made from the ITC buffer dialysate, and then pH corrected to 7.4. Protein and copper(II)ligand solutions were degassed with spinning for 10 minutes prior to loading (PAI-1 at 30uM, copper(II) 600-900 μM) to loading into the 1.394 mL ITC cell, and syringe, respectively. Copper(II)from the syringe entered the cell in 4 μL injections, at 120 second interval, totalling 45 injections. PAI-1 with 650 μM copper(II) datasets are shown as light gray circles, squares, and inverted triangles. PAI-1 with 750 μM copper(II) datasets are shown as circles, gray diamonds, and inverted triangles. The data were baseline corrected in NITPIC software, and a global fit to all of the data was performed using a two-site, nonsymmetric binding model in SEDPHAT software. The data, global fit and residuals are represented in GUSSI software via heats of injection (kcal/mol) as a function of copper(II)/PAI-1 molar ratio.

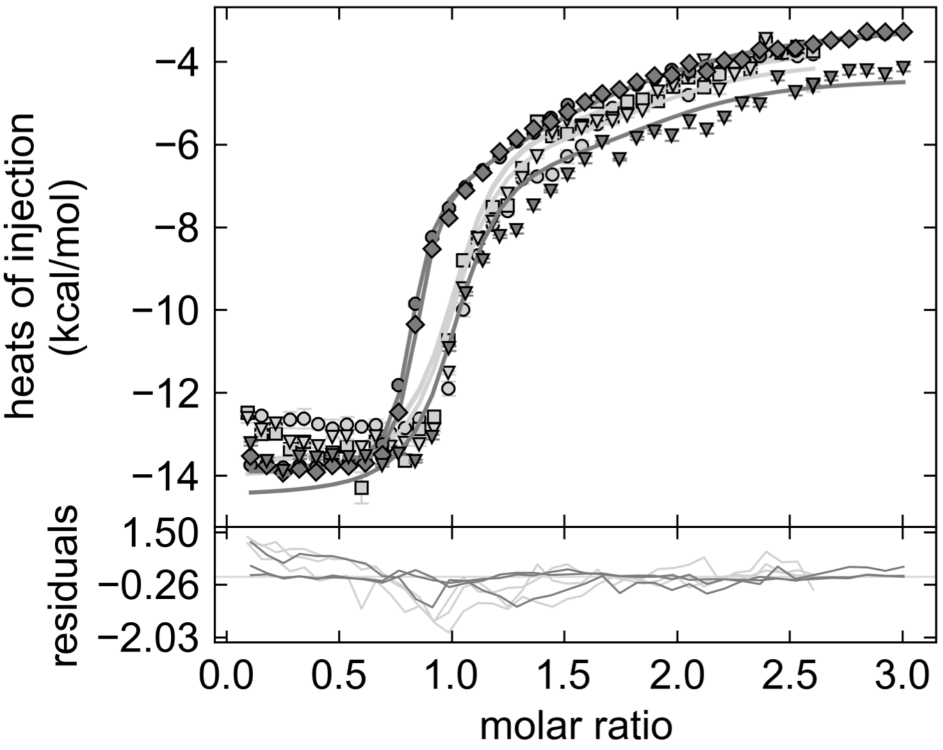

<u>Figure 4S</u>: ***ITC Measurements on Active H2AH3A PAI-1***
Active H2AH3AW175F PAI-1 was buffer exchanged into 100 mM MOPS, 250 mM $(NH_4)_2SO_4$, pH 7.4 at 10 °C using a PD10 column (GE Healthcare), followed by dialysis for 2 hours. A stock copper(II) solution concentration was verified using atomic absorption spectroscopy. Copper(II) solutions were made from the ITC buffer dialysate, and then pH corrected to 7.4. Protein and copper(II) ligand solutions were degassed with spinning for 10 minutes prior to loading (PAI-1 at 20 μ M, copper(II) 900-1200 μM) to loading into the 1.394 mL ITC cell, and syringe, respectively. Copper(II) from the syringe entered the cell in 4 μL injections, at 240 second interval, totalling 30 injections. PAI-1 replicates with 1200 μM copper(II) datasets are shown as gray circles and squares. PAI-1 replicates with 1350 μM copper(II) datasets are shown as light gray inverted triangles and triangles. The data were baseline corrected in NITPIC software, and a global fit to all of the data was performed using a one-site binding model in SEDPHAT software. The data, global fit and residuals are represented in GUSSI software via heats of injection (*kcal/mol*) as a function of copper(II)/PAI-1 molar ratio.

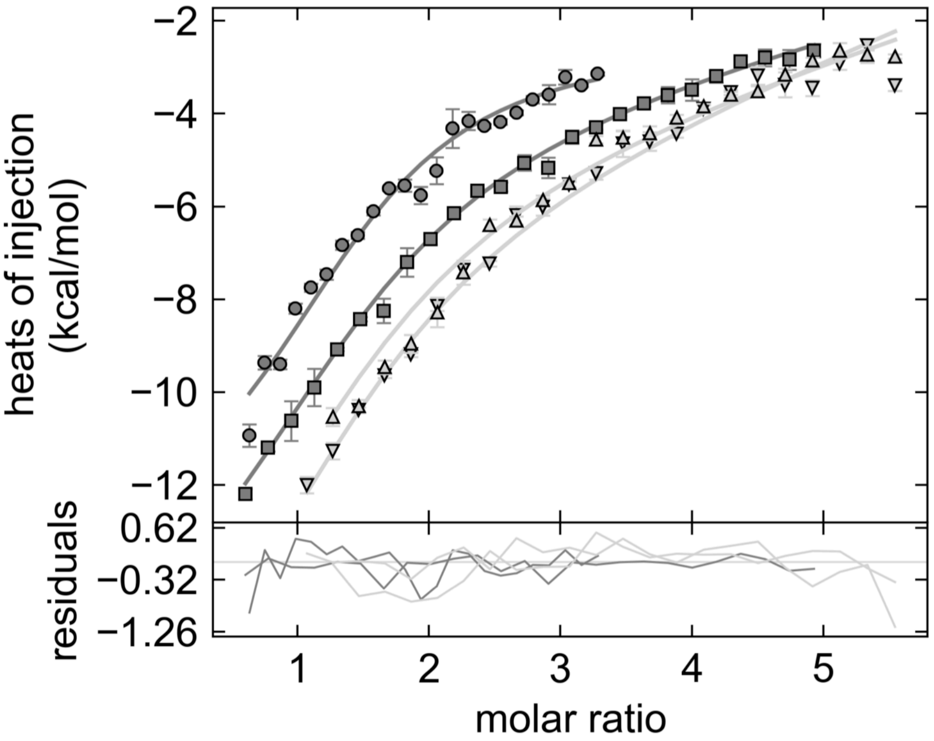

<u>Figure 5S</u>: *ITC Measurements on Latent H2AH3A PAI-1*

Latent H2AH3AW175F PAI-1 was buffer exchanged into 100 mM MOPS, 250 mM $(NH_4)_2SO_4$, pH 7.4 at 10 °C using a PD10 column (GE Healthcare), and dialysis for 2 hours. A stock copper(II) solution concentration was confirmed using atomic absorption spectroscopy. Copper(II) solutions were made from the ITC buffer dialysate, and then pH corrected to 7.4. Protein and copper(II) ligand solutions were degassed with spinning for 10 minutes prior to loading (PAI-1 at 20 µM, copper(II) 900-1200 µM) to loading into the 1.394 mL ITC cell, and syringe, respectively. Copper(II) from the syringe entered the cell in 4 µL injections, at 240 second interval, totalling 30 injections. PAI-1 with 1050 µM copper(II) datasets are shown as gray circles. PAI-1 with 1200 µM copper(II) replicate datasets are shown as light gray squares, inverted triangles, and triangles. The data were baseline corrected in NITPIC software, and a global fit to all of the data was performed using a one-site binding model in SEDPHAT software. The data, global fit and residuals are represented in GUSSI software via heats of injection (*kcal/mol*) as a function of copper(II)/PAI-1 molar ratio.

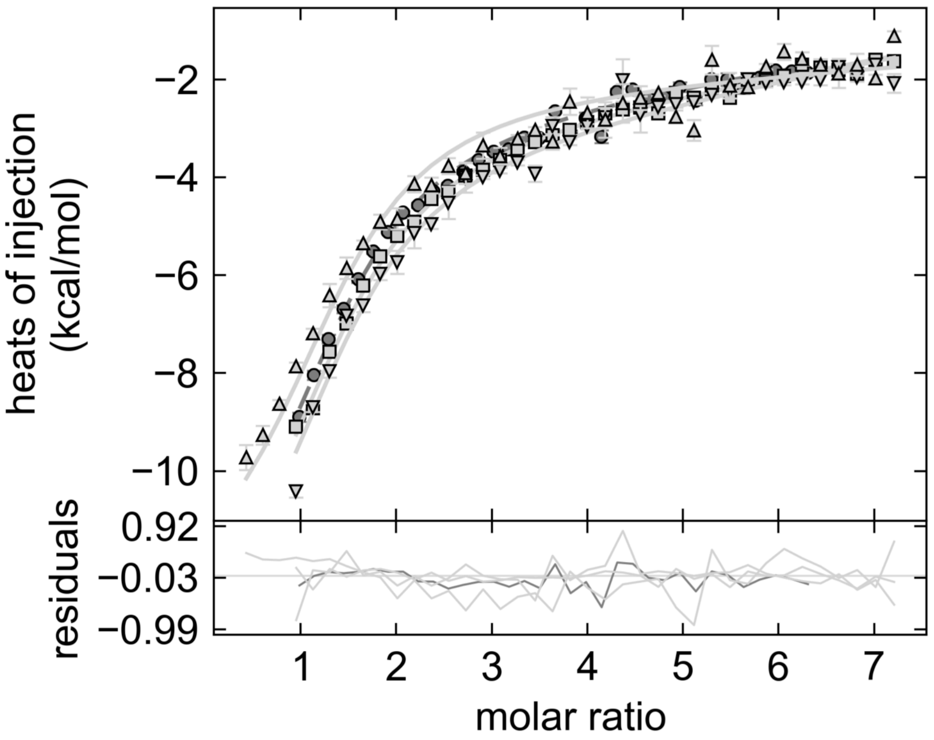

Supplement: Supplementary file 1 — Supplementary material 1 (PDF 1630 kb) [file 775_2017_1489_MOESM1_ESM.pdf]
